# Resveratrol Improves Bnip3-Related Mitophagy and Attenuates High-Fat-Induced Endothelial Dysfunction

**DOI:** 10.3389/fcell.2020.00796

**Published:** 2020-08-14

**Authors:** Chen Li, Ying Tan, Jiandi Wu, Qinghui Ma, Shuchang Bai, Zhangqing Xia, Xiaoliang Wan, Jianqiu Liang

**Affiliations:** ^1^Department of Cardiology, Foshan Hospital Affiliated with Southern Medical University (The Second People’s Hospital of Foshan), Foshan, China; ^2^Department of Critical Care Medicine, Nanfang Hospital, Southern Medical University, Guangzhou, China; ^3^Department of Oncology Hematology, Foshan Hospital Affiliated with Southern Medical University (The Second People’s Hospital of Foshan), Foshan, China

**Keywords:** resveratrol, mitochondria, oxidative stress, Bnip3, mitophagy

## Abstract

Statin treatment reduces cardiovascular risk. However, individuals with well-controlled low-density lipoprotein (LDL) levels may remain at increased risk owing to persistent high triglycerides and low high-density lipoprotein cholesterol. Because resveratrol promotes glucose metabolism and mitigates cardiovascular disorders, we explored its mechanism of protective action on high-fat-induced endothelial dysfunction. Human umbilical venous endothelial cells were treated with oxidized LDL (ox-LDL) *in vitro*. Endothelial function, cell survival, proliferation, migration, and oxidative stress were analyzed through western blots, quantitative polymerase chain reaction, ELISA, and immunofluorescence. ox-LDL induced endothelial cell apoptosis, proliferation arrest, and mobilization inhibition, all of which resveratrol reduced. ox-LDL suppressed the activities of mitochondrial respiration complex I and III and reduced levels of intracellular antioxidative enzymes, resulting in reactive oxygen species overproduction and mitochondrial dysfunction. Resveratrol treatment upregulated Bnip3-related mitophagy and prevented ox-LDL-mediated mitochondrial respiration complexes inactivation, sustaining mitochondrial membrane potential and favoring endothelial cell survival. We found that resveratrol enhanced Bnip3 transcription through hypoxia-inducible factor 1 (HIF1) and 5′ AMP-activated protein kinase (AMPK). Inhibition of AMPK and HIF1 abolished resveratrol-mediated protection of mitochondrial redox balance and endothelial viability. Together, these data demonstrate resveratrol reduces hyperlipemia-related endothelial damage by preserving mitochondrial homeostasis.

## Introduction

Hyperlipemia has been established as an independent risk factor for the development of atherosclerotic cardiovascular disease (Zeron and Albuquerque, 2019). Although statins have been used in clinical studies to reduce hyperlipemia, therapeutic resistance occurs in patients (Luirink et al., 2019), leading to additional studies on lipid-suppressing approaches for intervention. Although statins lower low-density lipoprotein (LDL) levels, they have little influence on high-density lipoprotein cholesterol (HDL-C) and triglycerides (Trieb et al., 2019). A remaining challenge, therefore, is to develop new drugs or compounds for controlling blood HDL-C levels. Resveratrol is a stilbenoid, which is produced by several plants in response to injury or when plants are under pathogen attack (Chen et al., 2019a; Wang et al., 2019). Many foods also contain resveratrol, including grapes, blueberries, raspberries, mulberries, and peanuts (Kim et al., 2019), and its protective actions include antioxidative and anti-inflammatory properties (Luo et al., 2019). At nutritionally relevant concentrations, resveratrol increases antioxidative enzyme gene expression and inhibits transcription of proinflammatory cytokines (Lejri et al., 2019; Rao et al., 2019). Previous studies have shown resveratrol promotes glucose metabolism as an adjunctive therapy for the management of diabetes-associated complications (Dludla et al., 2020). Additionally, resveratrol accelerates white adipocyte tissue browning (Li et al., 2020b) and fatty acid oxidation (Zhang et al., 2019d). However, few studies have explored the protective roles of resveratrol in hyperlipemia.

For the past few decades, most studies have focused on the roles of hyperlipemia in the heart, kidneys, and pancreas. Compared with these organs, endothelium is more vulnerable to hyperlipemia-triggered pathological injuries, including oxidative stress, metabolic disorder, cell senescence, and fibrosis (Wang et al., 2020a; Zhou and Toan, 2020). Additionally, owing to direct contact with blood, endothelium responds to blood composition alterations (Heusch, 2019; Wang et al., 2020b). Accordingly, hyperlipemia-mediated damage is likely to be seen in endothelium (Korbel et al., 2018). In addition, LDL is primarily degraded by endothelium through the LDL receptor, which is expressed on the surface of endothelium. Impaired endothelial function is associated with an increase in the blood LDL (Su et al., 2019). Based on this information, endothelium is an ideal barrier to regulate hyperlipemia. Several drugs targeting endothelium have been developed or investigated to attenuate hyperlipemia-related endothelial damage under metabolic disorder. Sitagliptin, a glucagon-like peptide analog, promotes upregulated vascular endothelial growth factor (VEGF) and enhances angiogenesis in diabetic rats (Khodeer et al., 2019). In a type-2 diabetes model, Empagliflozin inhibits oxidative stress and promotes endothelial cell migration and regeneration (Zhou et al., 2018c). Interestingly, resveratrol has been reported to inhibit hyperglycemia-mediated inflammatory “metabolic memory” in human retinal vascular endothelial cells (Zhang et al., 2015). However, no data are available to confirm whether resveratrol protects endothelial cells against high-fat-induced injuries.

At the sub-cellular level, hyperlipemia-induced injuries are usually caused by oxidative stress through free fatty acid (FFA) metabolism (Cesar et al., 2018). Compared with glucose, FFA metabolism consumes more oxygen, which is correlated with elevated reactive oxygen species (ROS) (Lee et al., 2019). In addition, hyperlipemia is always followed by metabolic reprogramming, which primarily uses FFAs rather than glucose as the energy substrates (Kowaltowski, 2019). Decreased glucose metabolism is accompanied with a decline in the production of antioxidative factors (Hysi et al., 2019). These two effects work together to augment intracellular oxidative stress, leading to endothelial cell dysfunction, including proliferation arrest, angiogenesis delay, mobilization inhibition, and apoptosis activation (Huang et al., 2018). A total of 85% intracellular ROS are generated at dysfunctional mitochondria through the tricarboxylic acid cycle and oxidative phosphorylation because of decreased expression or activity of mitochondrial respiration complexes (Silverblatt et al., 2019). Two strategies ameliorate mitochondrial ROS production: one is mediated through upregulation of endothelial antioxidative capacity, and the other is achieved through acceleration of dysfunction mitochondria clearance (Daiber and Chlopicki, 2020). Mitophagy, a selective form of autophagy, selectively targets damaged mitochondria and sustains mitochondrial homeostasis (Zhou et al., 2018d). The antioxidative property of mitophagy has been reported to play a role in the setting of diabetes, fatty liver disease, hypertension, and cardiac ischemia-reperfusion injury (Shi et al., 2018; Zhou et al., 2018a, 2019). Therefore, in this study, we investigated whether the ROS-reducing effect of resveratrol on high-fat-treated endothelial cells is mediated by mitophagy.

## Materials and Methods

### Cell Treatment and Transfection

HUVECs were cultured at 37°C in a humidified 5% CO_2_ environment (Zhang et al., 2019a). Culture medium was refreshed every 2–3 days. Cells were passed using trypsin-EDTA (Sigma, Steinheim, Germany) at 90–100%. HUVECs were used up to passage five. HUVEC stock solutions up to passage two were stored at 180°C in Dulbecco’s modified Eagle medium (DMEM) GlutaMAX containing 20% FBS and 10% DMSO (Sigma) (Zhao et al., 2019).

HUVECs were transiently transfected in triplicate with siRNA using Lipofectamine 2000 (Life Technologies, Frederick, MD, United States) according to the manufacturer’s instructions. Briefly, 8 mol/L siRNA and Lipofectamine transfection reagent (twice the amount of the total DNA quantity) were prepared separately in serum-free medium (Opti-MEM; Thermo Fisher Scientific, Waltham, MA, United States). Lipofectamine solution was added dropwise on DNA solution, and the mixture was incubated for 30 min at 37°C before gently added in each cell culture dish (Zhu et al., 2019). After 4 h, transfection medium was replaced with serum-free medium. After overnight recovery, cells were incubated with ox-LDL for the indicated time (Zarfati et al., 2019). Transient transfections were performed in triplicate wells in HUVECs and repeated n times.

### Real-Time Quantitative Polymerase Chain Reaction (RT-qPCR)

Total RNAs were extracted with TRIzol reagent (Strasbourg, France) according to the manufacturer’s instructions, and integrity was assayed by gel agarose electrophoresis. First-strand cDNA was synthesized with 1 μg of total RNA using iScriptTM cDNA Synthesis Kit (Bio-Rad, Hercules, CA, United States) according to the manufacturer’s instructions in a total volume of 20 μL (Zhang et al., 2019c). Transcript cDNA levels were analyzed in duplicate by RT-PCR performed with the CFX96 RT-PCR system (Bio-Rad) using SsoAdvanced^TM^ Universal SYBR^®^ Green Supermix (Bio-Rad), of which three contained 500 10^–9^ mol/L specific primers (Eurofins MWG Operon, Online Table I). Serial dilutions of pooled cDNA were used in each experiment to assess PCR efficiency. Gene expression was quantified relative to the geometric means of the housekeeping gene expression amplified in the same tube of investigated genes, and the ^Δ^ ^Δ^ Ct method was used to determine gene expression (Zhang et al., 2019b).

### ROS Measurement

HUVECs were suspended in DMEM and exposed to ox-LDL after transfection with Bnip3 siRNA. Total ROS production was measured by immunofluorescence with a 2′,7′-dichlorofluorescin diacetate Cellular ROS Detection Assay Kit (ab113851; Abcam, Cambridge, MA, United States) according to the manufacturer’s instructions (Quispe et al., 2019). Mitochondria-derived ROS levels in cardiomyocytes were measured using a mitochondrial superoxide indicator (MitoSOX^TM^ Red, M36008; Thermo Fisher Scientific) through immunofluorescence as previously described (Knani et al., 2019).

### Cell Survival Assay

A total of 50,000 cells/well were plated onto a 12-well plate. After 22 h, cells were replenished with fresh growth medium (Wolint et al., 2019), then 2 h later, cells were transfected with siRNA. Twenty-four hours after transfection, cell viability was measured through MTT assay as previously described (Trindade et al., 2019).

### Mitochondrial Isolation

Mitochondria were isolated from cells, digested with trypsin, homogenized with a glass/Teflon Potter Elvejhem homogenizer (Thomas Scientific, Swedesboro, NJ, United States), and then centrifuged at 800 × *g* for 10 min at 4°C. The supernatant was centrifuged at 8,000 × *g* for 10 min at 4°C, and the remaining supernatant was discarded. The pellet containing mitochondria was washed and centrifuged at 8,000 × *g* for 10 min at 4°C before resuspension (Zakeri et al., 2019). Mitochondrial protein concentration was determined by colorimetry using Bio-Rad protein assay dye reagent (500-0006; Bio-Rad).

### Cellular Respiration Assays

XPF extracellular flux analyzer (Seahorse Biosciences) was used for real = time analysis of the oxygen consumption rate (OCR) of intact cells according to the manufacturer’s instructions (van Duinen et al., 2019). Briefly, macrophages were seeded at 5 × 10^5^ cells/well in. After incubation with siRNA, mitochondrial respiration was detected according to the manufacturer’s instructions (Vijayan et al., 2019). Results were normalized to the actual cell count immediately after OCR recordings (Zhong et al., 2019).

### Immunostaining and Fluorescence Microscopy

Cells were quickly rinsed with PBS and fixed with 4% paraformaldehyde at room temperature for 15 min. Paraformaldehyde was then neutralized with NH_4_Cl for 15 min before cells were permeabilized with 0.5% Triton X-100 for 5 min, washed three times with PBS, rinsed with PBS 5% BSA for 40 min, then incubated overnight at 4°C with primary antibodies, followed by washes and incubation with Alexa Fluor^®^ 633 goat anti-rabbit IgG (H + L) (Molecular Probes, A21071; 1:1,000) for 1 h at room temperature (Xiao et al., 2019). Next, cells were rinsed with PBS, incubated with 5 μg/mL Hoechst 33342 (Sigma) for 5 min, washed again with PBS, and mounted with 15 μL Mowiol^®^ 4–88 (Calbiochem, San Diego, CA, United States). Cells were examined with a confocal microscope (Leica TCS-SP8 gated STED) (Yan et al., 2018). Alexa Fluor 633 was excited at 633 nm with a white light laser, and emission measured at 640–800 nm with a hybrid detector. Hoechst 33342 was excited by a 405-nm diode, and emission measured at 420–460 nm.

### Statistical Analysis

All data are shown as the mean ± standard error of the mean (SEM). A 2-sided, unpaired Student’s *T*-test between the groups for normal distributed variables and Mann-Whitney test for non-normal distributed variables were used for statistical testing. Differences across three or more groups were tested with ANOVA using Turkey’s multiple-comparison test or multiple Student’s *T*-test with Holm-Sidak corrections for multiple comparison. A *p*–value less than 0.05 was considered significant.

## Results

### Resveratrol Ameliorates Oxidized Low-Density Lipoprotein (ox-LDL)-Mediated Endothelial Dysfunction

**FIGURE 1:**
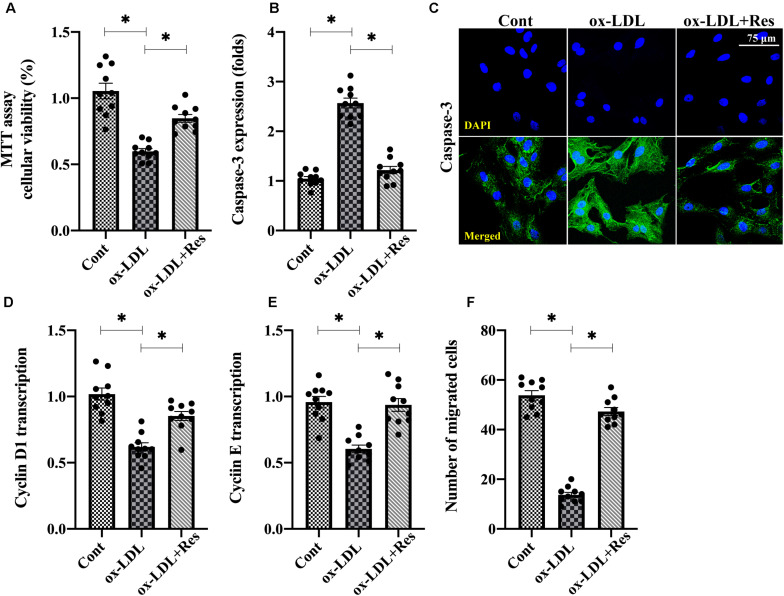
Resveratrol ameliorates ox-LDL-mediated endothelial dysfunction. **(A)** HUVECs were incubated with ox-LDL in the presence or absence of resveratrol. Cell viability was determined through MTT assay. **(B,C)** Immunofluorescence staining was used to observe the alterations of caspase-3 in endothelial cells under ox-LDL treatment. **(D,E)** RNA was collected from HUVECs after treatment with ox-LDL in the presence or absence of resveratrol. Then, transcription of cyclin D1 and cyclin **(E)** were measured to reflect endothelial cell proliferation. **(F)** Transwell assay was used to detect endothelial cell migratory response. The number of migrated endothelial cells was recorded. *p < 0.05.

To understand the role of resveratrol in the dysfunction of high-fat-mediated endothelial cells, human umbilical venous endothelial cells (HUVECs) were cultured with ox-LDL in the presence or absence of resveratrol. Then, endothelial cell viability, proliferation, and mobilization were determined. Figure 1A shows that compared with the control group, MTT assay demonstrated ox-LDL reduced endothelial viability, whereas this alteration was corrected by resveratrol. Immunofluorescence assay for caspase-3 also showed that the expression of caspase-3 was rapidly increased or drastically inhibited by ox-LDL or resveratrol, respectively (Figures 1B,C). Therefore, these results indicate that resveratrol sustains endothelial viability in the setting of hyperlipemic stress. RNA analysis of cyclin D1 and cyclin E also illustrated that endothelial cell proliferation rate was impaired by ox-LDL (Figures 1D,E). Interestingly, resveratrol treatment upregulated the transcription of cyclin D1 and cyclin E (Figures 1D,E), suggesting that resveratrol attenuates high-fat-induced endothelial growth arrest. With respect to endothelial mobilization, Transwell assay demonstrated that the number of migrated endothelial cells was downregulated after exposure to ox-LDL (Figure 1F). Resveratrol pretreatment maintained endothelial cell mobilization through increasing the number of migrated cells. Thus, these data indicate that endothelial mobility is preserved by resveratrol in the setting of hyperlipemia.

### Oxidative Stress Is Inhibited by Resveratrol in ox-LDL-Treated Endothelial Cells

**FIGURE 2:**
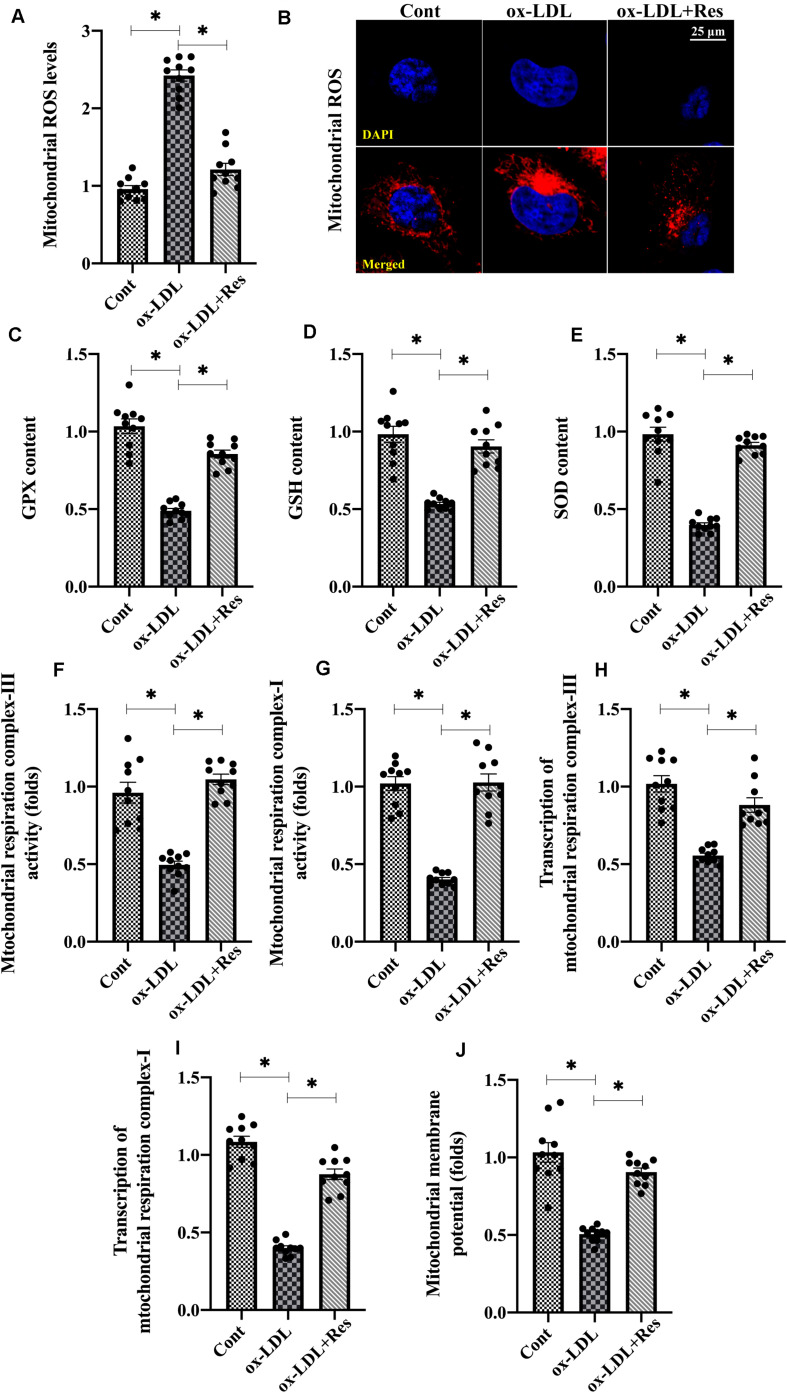
Oxidative stress is inhibited by resveratrol in ox-LDL-treated endothelial cells. **(A,B)** Immunofluorescence assay for mitochondrial ROS in endothelial cells treated with ox-LDL in the presence or absence of resveratrol. **(C–E)**. ELISA was used to evaluate the activities of GSH, SOD, and GPX in endothelial cells. **(F,G)**. The activities of mitochondrial respiration complex I and III were measured through ELISA. **(H,I)** qPCR was used to analyze the transcription of mitochondrial respiration complex I and III. **(J)** JC-1 probe was used to stain mitochondrial membrane potential. The red-to-green fluorescence intensity was used to quality mitochondrial membrane potential. *p < 0.05.

To explain the protective effects underlying resveratrol-sustained endothelial viability, proliferation and mobilization, endothelial redox status, mitochondrial oxidative stress was analyzed. First, the levels of mitochondrial ROS were elevated in response to ox-LDL treatment (Figures 2A,B); resveratrol ameliorated this effect, confirming its antioxidative property. Antioxidative factors such as GSH, SOD, and GPX neutralized upregulated ROS. However, GSH, SOD, and GPX transcriptions were drastically downregulated in ox-LDL-treated endothelial cells (Figures 2C–E), although resveratrol could restore their expressions.

Mitochondrial ROS are primarily generated by mitochondrial respiration complexes, especially mitochondrial respiration complex I and III (DeLeon-Pennell et al., 2018). Using ELISA, we found that the mitochondrial respiration complex I and III activities decreased with ox-LDL treatment, whereas this phenotypic alteration could be normalized with resveratrol (Figures 2F,G). Mitochondrial respiration complex I and III transcriptions were also downregulated or upregulated by ox-LDL or resveratrol, respectively (Figures 2H,I). Owing to mitochondrial ROS overload, mitochondrial function, which is evaluated by the mitochondrial membrane potential, was also blunted in ox-LDL-treated endothelial cells (Figure 2J). However, resveratrol pretreatment stabilized mitochondrial membrane potential in the presence of ox-LDL (Figure 2J). Together, our results indicate that mitochondrial oxidative stress, which is triggered by ox-LDL, could be repressed by resveratrol in endothelial cells.

### Resveratrol Activates Bnip3-Related Mitophagy in Endothelial Cells in the Presence of ox-LDL

**FIGURE 3:**
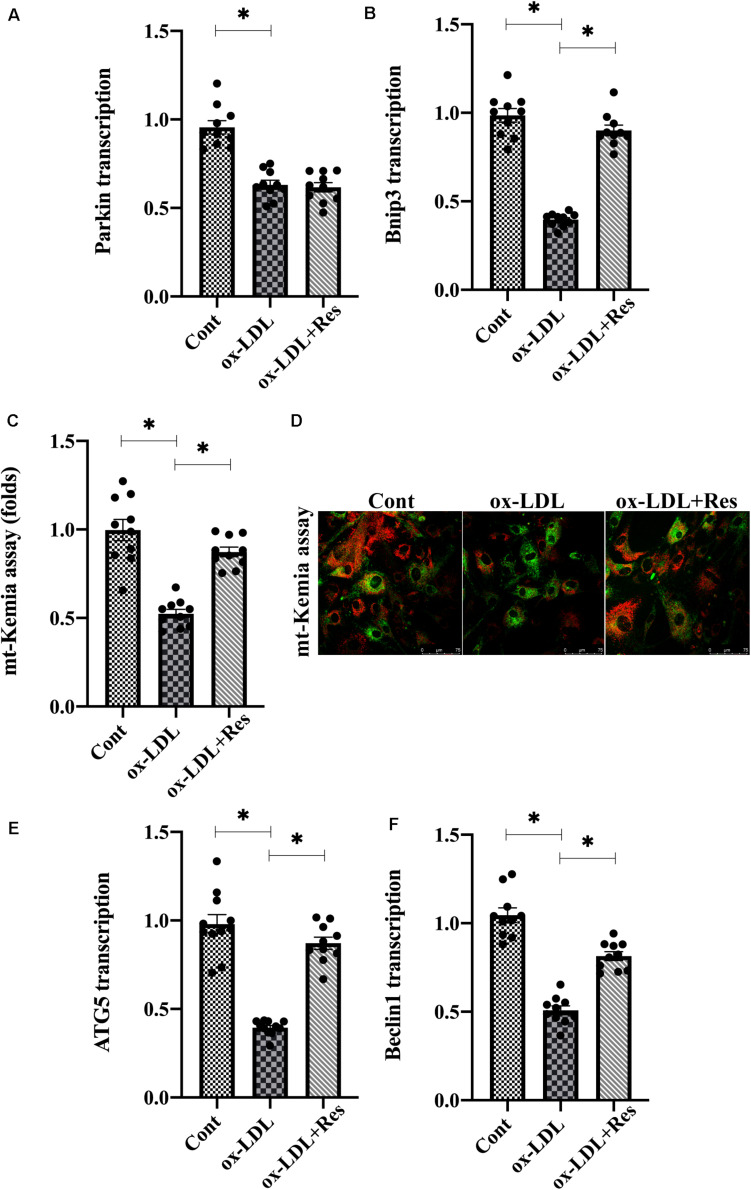
Resveratrol activates Bnip3-related mitophagy in endothelial cells in the presence of ox-LDL. **(A,B)** After treatment with ox-LDL, RNA in endothelial cells was collected and Parkin and Bnip3 transcription was measured. **(C,D)** Endothelial mitophagy was detected through mt-Kemia. The acid mitochondria number was recorded to reflect the activity of mitophagy. **(E,F)** ATG5 and Beclin1 transcription was determined through qPCR. Endothelial cells were treated with ox-LDL in the presence or absence of resveratrol. *p < 0.05.

As a compensatory repairing system, mitophagy selectively guides dysfunction to be degraded by lysosome contributing to intracellular redox balance. Therefore, we investigated whether resveratrol regulated endothelial mitochondrial oxidative stress through mitophagy. Mitophagy is activated by several adaptors, including Parkin and BCL2/adenovirus E1B 19-kDa protein-interacting protein 3 (Bnip3) (Zhou et al., 2018b). Interestingly, ox-LDL treatment repressed Parkin and Bnip3 expression in endothelial cells (Figures 3A,B). Resveratrol treatment reversed Bnip3 expression but had a slight effect on Parkin expression in ox-LDL-treated endothelial cells (Figures 3A,B), suggesting that endothelial mitophagy could be activated by resveratrol in a manner dependent on Bnip3. To demonstrate the promotive action by resveratrol on mitophagy, mt-Kemia probe, an acid mitochondria indicator, was added into endothelial cell medium. Under normal conditions, mitophagy is moderate and, thus, parts of acid mitochondria could be detected (Figures 3C,D). After exposure to ox-LDL, the acid mitochondrial number was reduced, and this trend could be corrected by resveratrol (Figures 3C,D). In addition, RNA analysis demonstrated that ATG5 and Beclin1, the mitophagy markers, were transcriptionally inhibited by ox-LDL (Figures 3E,F). However, resveratrol treatment upregulated the ATG5 and Beclin1 RNA expression (Figures 3E,F), reconfirming a contributory action underlying resveratrol on endothelial mitophagy in the presence of ox-LDL.

### Inhibition of Bnip3-Related Mitophagy Suppresses Resveratrol-Induced Protection on Mitochondrial Homeostasis

**FIGURE 4:**
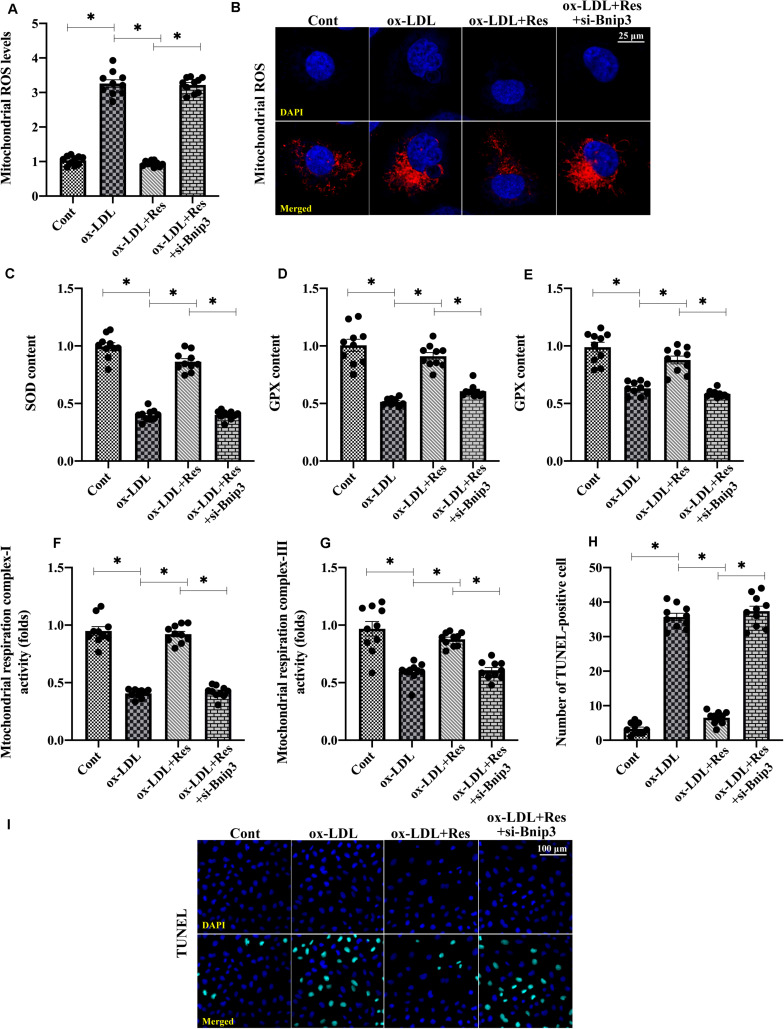
Inhibition of Bnip3-related mitophagy suppresses resveratrol-induced protection on mitochondrial homeostasis. **(A,B)** Bnip3 siRNA was transfected into endothelial cells before treated with resveratrol. Then, mitochondrial ROS levels were measured through immunofluorescence. **(C–E)** ELISA was used to evaluate GSH, SOD, and GPX activity in endothelial cells. **(F,G)** Mitochondrial respiration complex I and III activities were measured through ELISA. **(H,I)** TUNEL staining was used to quantify apoptotic endothelial cells in response to Bnip3 knockdown. *p < 0.05.

To understand whether Bnip3-related mitophagy is required for resveratrol-induced mitochondrial protection, we silenced Bnip3 in resveratrol-treated endothelial cells. Then, mitochondrial function and redox biology were analyzed. Figures 4A,B shows that compared with the control group, mitochondrial ROS production was elevated by ox-LDL. Although resveratrol suppressed mitochondrial ROS generation, this effect was abolished by Bnip3 siRNA (Figures 4A,B). The levels of antioxidative factors, such as GSH, SOD, and GPX, were downregulated by ox-LDL and reversed to near-normal levels after resveratrol pretreatment (Figures 4A–E). Interestingly, with the loss of Bnip3-related mitophagy, resveratrol failed to upregulate intracellular antioxidative factors in ox-LDL-treated endothelial cells (Figures 4C–E). In addition, ox-LDL repressed mitochondrial respiration complex I and III activities. Resveratrol corrected this alteration in a manner dependent on Bnip3 (Figures 4F,G), suggesting that Bnip3-mediated mitophagy may promote mitochondrial respiration. Last, TUNEL staining was used to confirm whether Bnip3-related mitophagy was necessary for endothelial cell survival. Figures 4H,I shows that compared with the control group, ox-LDL increased the ratio of TUNEL-positive endothelial cells. Although resveratrol could inhibit ox-LDL-mediated endothelial cell death, this protective action was undetectable in endothelial cells transfected with Bnip3 siRNA (Figures 4H,I). Together, our results indicate that Bnip3-related mitophagy is required for resveratrol-mediated mitochondrial protection in ox-LDL-treated endothelial cells.

### Resveratrol Regulates Bnip3 Through Hypoxia-Induced Factor 1 (HIF1) and 5′ AMP-Activated Protein Kinase (AMPK)

**FIGURE 5:**
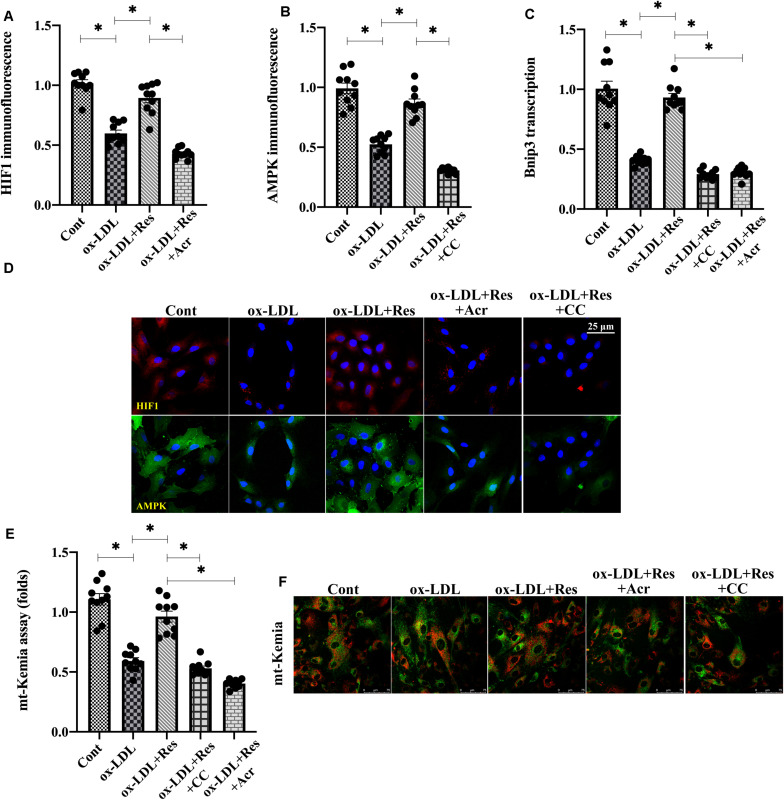
Resveratrol regulates Bnip3 through the HIF1 and AMPK. **(A–C)** Immunofluorescence assay for HIF1 and AMPK in endothelial cells treated with ox-LDL in the presence or absence of resveratrol. Acriflavine (Acr) and compound c (CC), the antagonists of HIF1 and AMPK, respectively, were incubated with endothelial cells before resveratrol treatment. **(D)** qPCR was used to determine the Bnip3 alteration in endothelial cells treated with ox-LDL, resveratrol, Acr, or CC. **(E,F)** Endothelial mitophagy was detected through mt-Kemia. The acid mitochondria number was recorded to reflect mitophagy activity. *p < 0.05.

Previous studies have reported that Bnip3 is primarily regulated by two pathways: one is HIF1 (Guo et al., 2001) and the other is AMPK (Park et al., 2013). Experiments were conducted to understand whether these two pathways were activated by resveratrol, promoting Bnip3-related mitophagy. Immunofluorescence assay demonstrated that both HIF1 and AMPK were downregulated in response to ox-LDL treatment (Figures 5A–C). Interestingly, resveratrol treatment was associated with an increase in HIF1 and AMPK levels (Figures 5A–C), confirming our hypothesis that both HIF1 and AMPK could be positively regulated by resveratrol. To investigate whether increased HIF1 and AMPK were implicated in resveratrol-induced Bnip3 upregulation, acriflavine (Acr) and compound c (CC), the antagonists of HIF1 and AMPK, respectively, were incubated with endothelial cells before resveratrol treatment. Then, Bnip3 transcription and mitophagy activity were remeasured. Figure 5D shows that resveratrol treatment sustained the transcription of Bnip3 in ox-LDL-treated endothelial cells. However, once supplemented with either Acr or CC, the transcription of Bnip3 was downregulated (Figure 5D), suggesting that inhibition of HIF1 or AMPK could abolish the regulatory effects by resveratrol on Bnip3. mt-Kemia assays showed resveratrol upregulated the number of acid mitochondria in the presence of ox-LDL (Figures 5E,F), indicative of mitophagy activation in response to resveratrol. However, treatment with Acr or CC reduced acid mitochondrial content in resveratrol-treated endothelial cells, suggesting that HIF1 blockade of AMPK is followed by mitophagy inactivation. Taken together, our results indicate that resveratrol upregulates Bnip3-related mitophagy through HIF1 and AMPK.

## Discussion

Oxidized LDL contributes to endothelial dysfunction, which is followed by vascular inflammation, smooth muscle proliferation, plaque formation, luminal stenosis, and other pathological alterations involved in the development of atherosclerosis. Statin treatment results in significant reductions in cardiovascular risk; however, individuals with well-controlled LDL levels may remain at increased risk owing to persistent high triglycerides and low HDL (Seidel et al., 2019). Therefore, an urgent need exists for hyperlipemia management. In the present study, we found that resveratrol improved endothelial function through sustaining endothelial cell viability, proliferation, and mobilization (Rusnati et al., 2019). Resveratrol treatment activated HIF1 and AMPK pathways, contributing to Bnip3 upregulation and mitophagy activation. Subsequently, Bnip3-related mitophagy attenuated oxidative stress and sustained mitochondrial function in the setting of hyperlipemia. To our knowledge, these data provide the first evidence for the use of resveratrol in preventing high-fat-mediated endothelial dysfunction. Our results identify Bnip3-related mitophagy as a primary protective mechanism responsible for resveratrol-mediated endothelial protection.

Most previous in-depth studies have explored the roles of resveratrol in cardiovascular disorders. For example, resveratrol enhances the expression of Nrf2 in myocardium and, thus, alleviates myocardial ischemia-reperfusion injury (Schreiber et al., 2019; Xu et al., 2019). Administration of resveratrol is shown to retard the progression of pulmonary arterial hypertension (Ferreira et al., 2019). Treatment with resveratrol attenuates isoprenaline-related cardiotoxicity in Wistar rats (Sammeturi et al., 2019). Resveratrol prevents ventricular hypertrophy (Chelladurai et al., 2019) and cardiac remodeling following chronic kidney disease (Li et al., 2020a). Resveratrol also helps to protect aortic valve stenosis (Samiei et al., 2019), septic cardiomyopathy (Liang et al., 2019), diabetic cardiomyopathy (Hoseini et al., 2019), and heart failure (Algieri et al., 2019). Most associated studies have focused on the influence of resveratrol on hyperlipemia-related cardiomyocyte damage or metabolic reprogramming, but not on high-fat-related endothelial dysfunction. Our data support that resveratrol, leading to improved endothelial function in the setting of hyperlipemia, may open a therapeutic window for the treatment of atherosclerosis.

At the molecular level, two mechanisms involved in how resveratrol attenuates hyperlipemia-related cardiomyocyte injury have been reported: one is driven by upregulation of antioxidative factors and the other involves downregulation of pro-inflammation cytokines. For example, resveratrol attenuates lipid peroxidation (Jalili et al., 2019) through modulation of several antioxidative signaling pathways such as Nrf2 (Zhuang et al., 2019), Sirt1 (Liu et al., 2019a), Akt/mTOR (Radwan and Karam, 2020), and ERK1/2 (Fathalipour et al., 2019). In addition, the mRNA expression of inflammatory cytokines in diabetic mice are largely inhibited by resveratrol (Xing et al., 2020). Inflammation-related signaling pathways, such as NF-κB (Ma et al., 2020), Smad2/3 (Zou et al., 2019), and HSP70 (Khafaga et al., 2019), are also blocked by resveratrol. In the present study, we found that resveratrol modulated mitochondrial ROS production through affecting mitochondrial respiration complex I and III activities. This provides novel insight into the regulatory actions of resveratrol on redox biology. Similar to our results, previous studies have also reported the involvement of resveratrol in mitochondrial homeostasis (Nwadozi et al., 2019). For example, resveratrol improves mitochondrial ATP generation through the AMPK pathway in the ischemic brain (Pineda-Ramirez et al., 2020). Mitochondrial biogenesis is partly enhanced by resveratrol through the miR-22/Sirt1 signaling pathway (Mao et al., 2019). Mitochondrial calcium homeostasis and mitochondrial potential stabilization are also under the control of resveratrol (Algieri et al., 2019). Mitochondrial morphological alterations, such as mitochondrial fission and fusion, are also balanced by resveratrol in different types of cells, such as hepatocytes (Chen et al., 2019b), cardiomyocytes (Lu et al., 2019), and endothelium (Yu et al., 2019).

Our data identified Bnip3-related mitophagy was activated by resveratrol and attenuated ox-LDL-induced mitochondrial damage as well as oxidative stress. Interestingly, after exposure to ox-LDL, both Parkin and Bnip3 were downregulated, whereas resveratrol upregulated Bnip3 transcription. This finding is consistent with a previous study that Parkin-dependent mitophagy usually works in neurodegenerative disease, whereas Bnip3-related mitophagy affects metabolic disorders such as fatty liver disease and diabetes (Li et al., 2018). Additionally, we demonstrated resveratrol upregulated Bnip3 transcription through HIF1 and AMPK. AMPK is a sensor of cellular energy status. Increased AMPK is able to upregulate gene transcription, including Bnip3. This phenomenon has been observed in diabetic nephropathy (Liu et al., 2019b) and muscle atrophy (Bak et al., 2019). HIF1 is a hypoxia-activated transcriptional factor (Martinez-Outschoorn et al., 2010), whereas Bnip3 is a hypoxia-related gene (Guo et al., 2001). Additional studies have reported the causal relationship between HIF1 activation and Bnip3 upregulation (Park et al., 2013). In the present study, we found that both AMPK and HIF1 were employed by resveratrol to enhance Bnip3-related mitophagy. The interlinked signaling pathways of Bnip3-related mitophagy in response to resveratrol treatment would offer new targets for therapeutic approaches of endothelial protection in the setting of hyperlipemia.

Together, using biochemical approaches and genetic deletion *in vitro*, we identify a potentially novel pathway by which resveratrol attenuates high-fat-induced endothelia dysfunction dependently of the Bnip3-related mitophagy. However, clinical data and animal studies are necessary to support our findings.

## Data Availability Statement

The raw data supporting the conclusions of this article will be made available by the authors, without undue reservation.

## Author Contributions

CL and YT contributed to the study concepts, experiment performance, and the data acquisition. JW, QM, and SB contributed to manuscript preparation and the data analysis. ZX and XW contributed to statistical analysis and manuscript review. JL was involved in manuscript editing. All authors contributed to the article and approved the submitted version.

## Conflict of Interest

The authors declare that the research was conducted in the absence of any commercial or financial relationships that could be construed as a potential conflict of interest.
