# Expression of Concern: Resveratrol improves Bnip3-related mitophagy and attenuates high-fat-induced endothelial dysfunction

**DOI:** 10.3389/fcell.2022.1014318

**Published:** 2022-08-23

**Authors:** Frontiers Editorial Office

Following publication, the publisher has uncovered conclusive evidence that false identities were used as peer reviewers for this article. These reviewers were not suggested by the authors. These peer reviewers have now been removed.

This article is currently under post publication assessment. This expression of concern has been posted while Frontiers awaits the outcome of this assessment and will then be updated accordingly.

